# SHOX2 DNA Methylation is a Biomarker for the diagnosis of lung cancer based on bronchial aspirates

**DOI:** 10.1186/1471-2407-10-600

**Published:** 2010-11-03

**Authors:** Bernd Schmidt, Volker Liebenberg, Dimo Dietrich, Thomas Schlegel, Christoph Kneip, Anke Seegebarth, Nadja Flemming, Stefanie Seemann, Jürgen Distler, Jörn Lewin, Reimo Tetzner, Sabine Weickmann, Ulrike Wille, Triantafillos Liloglou, Olaide Raji, Martin Walshaw, Michael Fleischhacker, Christian Witt, John K Field

**Affiliations:** 1 Universitätsklinik und Poliklinik für Innere Medizin I, Universitätsklinikum Halle (Saale), Halle, Germany; 2 Metanomics Health GmbH, Berlin, Germany; 3 Epigenomics AG, Berlin, Germany; 4 Theracode GmbH, Mainz, Germany; 5 Bavarian Nordic GmbH, Berlin, Germany; 6 Medizinische Klinik m.S. Infektiologie und Pneumologie, Charité-Universitätsmedizin, Berlin, Germany; 7 Berlin Institute of Technology, Institute for Biotechnology, Berlin, Germany; 8 Roy Castle Lung Cancer Research Programme, University of Liverpool Cancer Research Centre, Liverpool L3 9TA, UK; 9 Liverpool Heart & Chest Hospital NHS Trust, Thomas Drive, Liverpool L14 3PE, UK; 10 Medizinische Klinik m.S. Hämatologie Onkologie, Charité-Universitätsmedizin, Berlin, Germany

## Abstract

**Background:**

This study aimed to show that SHOX2 DNA methylation is a tumor marker in patients with suspected lung cancer by using bronchial fluid aspirated during bronchoscopy. Such a biomarker would be clinically valuable, especially when, following the first bronchoscopy, a final diagnosis cannot be established by histology or cytology. A test with a low false positive rate can reduce the need for further invasive and costly procedures and ensure early treatment.

**Methods:**

Marker discovery was carried out by differential methylation hybridization (DMH) and real-time PCR. The real-time PCR based HeavyMethyl technology was used for quantitative analysis of DNA methylation of SHOX2 using bronchial aspirates from two clinical centres in a case-control study. Fresh-frozen and Saccomanno-fixed samples were used to show the tumor marker performance in different sample types of clinical relevance.

**Results:**

Valid measurements were obtained from a total of 523 patient samples (242 controls, 281 cases). DNA methylation of SHOX2 allowed to distinguish between malignant and benign lung disease, i.e. abscesses, infections, obstructive lung diseases, sarcoidosis, scleroderma, stenoses, at high specificity (68% sensitivity [95% CI 62-73%], 95% specificity [95% CI 91-97%]).

**Conclusions:**

Hypermethylation of SHOX2 in bronchial aspirates appears to be a clinically useful tumor marker for identifying subjects with lung carcinoma, especially if histological and cytological findings after bronchoscopy are ambiguous.

## Background

Lung cancer is the second most common cancer in both men and women representing about 15% of all cancer diagnoses [1]. In the absence of screening, lung cancer patients either exhibit symptoms or are accidentally diagnosed by clinical imaging performed for other indications. Patients suspected of having malignant lung disease usually undergo clinical investigation (workup) including CT-scanning of the thorax and bronchoscopy, which is mainly undertaken in those individuals with central tumours. The latter is the method of choice for confirming the diagnosis of a suspected lung neoplasm by pathological assessment of tissue or a cytological specimen obtained during the procedure.

The prevalence of lung cancer in this group of patients investigated for suspected lung cancer is approximately 30-40% (personal communication Prof. Field). Establishing a final diagnosis after the first bronchoscopy fails in about half of these patients [2], triggering additional invasive diagnostic procedures. Even when signs, symptoms and radiological findings are such that the clinical diagnosis of malignant lung disease appears obvious, it often takes considerable effort and invasive procedures to obtain tissue material suitable for definitively confirming the presence of malignant disease.

Ambiguous results (i.e. the presence of malignancy cannot be confirmed) following bronchoscopy are not uncommon, e.g. because the tumor is not visible endoscopically and cells obtained by brushing or aspiration do not allow the pathologist to confirm or exclude malignancy. In these cases, several additional diagnostic procedures are available, each with its own pros and cons:

• Histology from needle biopsy (transbronchial or transthoracic) or surgical intervention is the gold standard for establishing the diagnosis of malignant disease. These procedures are invasive and may cause complications like pneumothorax and bleedings [3,4].

• Repeated CT-scan after about 12 weeks is able to detect growth of a lesion. This is a means for increasing the specificity of CT-scanning for detecting malignancy, but it is only a surrogate marker and may lead to a delay in establishing the diagnosis.

• PET scanning is clinically valuable for identifying areas of hypermetabolism. It can only detect lesions with a diameter of about 1 cm or more and is currently an expensive investigative method [5]. Additionally, increased metabolism is not a cancer specific phenomenon.

Biomarkers have great potential for improving the management of lung cancer in clinical routine. So far, several biomarkers from various sources such as genetics, proteomics, and epigenetic approaches are in use for clinical research purposes [6-8]. The analysis of DNA methylation biomarkers is an emerging field that provides promising potential for improving the clinical process of lung cancer diagnosis [9-13]. Methylation of DNA is an important epigenetic process involved in fundamental biological events such as development and cell differentiation [14]. Aberrant DNA methylation has been reported to play a major role in carcinogenesis [15], suggesting that DNA methylation analysis may be a valuable source for cancer biomarkers [16].

In the presented study, SHOX2 methylation was identified as a biomarker capable of reliably differentiating between lung tumor and normal tissues. This genome wide discovery approach was carried out using differential methylation hybridization (DMH) technology [17]. A real-time PCR based assay for highly sensitive and accurate quantification of methylated SHOX2 copies in a background of unmethylated DNA was developed. This assay was then used to quantify the SHOX2 DNA methylation in bronchial aspirates from 523 patients to investigate its ability to identify patients with lung cancer in a population of individuals with suspected lung cancer. A calibrator, a DNA sample with known methylation, was used in order to normalize for lot-to-lot and site-to-site variability and therefore to allow for routine clinical usage of the test. SHOX2 DNA methylation was shown to reliably detect cancer patients at high specificity in a group of patients with benign lung diseases, i.e. abscesses, infections, obstructive lung diseases, sarcoidosis, scleroderma and stenoses, who underwent the same clinical workup for suspected lung cancer.

## Methods

### Patients

Bronchial aspirates were collected at two medical centres with appropriate written consent under approval of the local ethics committees. 246 fresh-frozen specimens were provided by the Charité University Hospital (Berlin, Germany); 388 Saccomanno-fixed specimens came from the Roy Castle Lung Cancer Research Program [18] (Cancer Research Centre, Liverpool, UK). All patients donating samples were investigated for suspected lung cancer in the respective clinics. 141 of the fresh-frozen sample specimens showed cytology negative result after bronchoscopy. Samples from 523 patients passed the sample quality control acceptance criterion as described in chapter 'Data and Statistical Analysis' and were suited for analyzing the SHOX2 DNA methylation. The characteristic of this population is described in more detail in Table 1.

**Table 1:** Characteristics of the patient population.

	Total	Cases	Controls
			
Age	523 (100%)	281 (100%)	242 (100%)
≤ 50 Years	59 (11%)	15 (5%)	44 (18%)
51-60 Years	110 (21%)	59 (21%)	51 (21%)
>60 Years	344 (66%)	206 (73%)	138 (57%)
Unknown	10 (2%)	1 (0%)	9 (4%)
Median Age	66	67	65
Age Range	23-90	37-90	23-85
**Smoking Habits**			

Non-smokers	90 (17%)	21 (7%)	69 (29%)
Smokers (Current and Former)	320 (61%)	213 (76%)	107 (44%)
Unknown Smoking Status	113 (22%)	47 (17%)	66 (27%)
Range Packs/Years (Smokers only)	0-171	0-171	0-141
Median Packs/Years (Smokers only)	32	40	10
Mean Packs/Years (Smokers only)	34	42	22
**Histology Subtype**			

Squamous Cell Carcinoma	x	103 (37%)	x
Adenocarcinoma	x	109 (39%)	x
NSCLC NOS	x	37 (13%)	x
SCLC	x	29 (10%)	x
Other/Unknow	x	3 (1%)	x
**Stage (UICC)**			

I	x	59 (21%)	x
II	x	43 (15%)	x
III	x	108 (38%)	x
IV	x	62 (22%)	x
Unknown	x	9 (3%)	x

Clinical data of the 523 analyzed patient samples (281 [54%] Cases, 242 [46%] Controls). 111 samples (68 Saccomanno-fixed cases, 4 fresh-frozen cases, 34 Saccomanno-fixed controls, 5 fresh-frozen controls) failed the sample quality control because their DNA yield was too low. They were excluded from analysis and are not included in this table.

Bronchial samples were collected during bronchoscopy by aspiration with a flexible bronchoscope from the region of the suspicious lesion after injecting 10-20 ml of isotonic saline solution and prior to starting any cancer specific treatment, if applicable.

The diagnosis of bronchial carcinoma was confirmed by one or more of the following approaches: cytology or histology from biopsy or surgery specimen. Cases have been selected to include a high number of stage I or II (UICC) disease and to represent the main NSCLC histology types. Patients that underwent workup for suspected lung cancer in the same time period and at the same clinics, did not show any evidence of malignant lung disease and had a minimum lung cancer free survival of 12 months were considered as the 'control' group of patients for this study.

Samples fixed with Saccomanno's reagent were stored at room temperature for up to 12 years. The Median age of Cases and Controls was 67 and 65 years respectively.

For frozen storage, the unfixed aspirates were centrifuged according to clinical routine procedures and the pellets were stored at -80°C for up to 7 years.

### Differential Methylation Hybridization (DMH)

Differential methylation hybridization (DMH) for genome-wide DNA methylation profiling using a CpG island microarray representing more than 50,000 CpG-rich DNA fragments was carried out as previously described [19].

### Sample and Calibrator Preparation

A calibrator sample with known methylation level (1%) and known total DNA (50 ng) content was prepared by mixing bisulfite converted DNA from sperm with bisulfite converted methylated DNA. DNA extraction from sperm and the bisulfite conversion of sperm DNA and methylated DNA was carried out as previously described [20].

DNA from bronchial aspirates was isolated by means of the QIAamp^® ^DNA Micro Kit (Qiagen) using a modified tissue protocol (Kit handbook). Bisulfite conversion of DNA was performed using the EpiTect Kit (Qiagen).

### Real-Time PCR

*Real-time PCR (SYBR Green Assay) for the Analysis of DNA from Tissue Specimens. *PCR was carried out using the QuantiTect Multiplex Kit (Qiagen, Hilden, Gemany) with 0.3 μM of each primer (forward: GTTTTTTGGATAGTTAGGTAAT, reverse: CCTCCTACCTTCTAACCC), 1 μM blocker (TAATTTTTGTTTTGTTTGTTTGATTGGGGTTGTATGA-SpacerC3), 10 ng DNA template (quantified via UV spectrophotometry) and 1:40,000 diluted SYBR Green I DNA dye (Biozym Scientific, Oldendorf, Germany) in 20 μl per reaction. Real-time PCR was performed on the LC480 platform (Roche Diagnostics, Mannheim, Germany) with the following program: 95°C/10 min, 45 cycles with 95°C/10 sec, 56°C/30 s, 72°C/10 and 82.5°C/5 s (detection step).

*Real-time PCR (Probe Assay) for the Analysis of DNA from Lavage Specimens. *Real-time PCR assays were comprised of two independent reactions: a total quantification assay for quantification of total input DNA and an HM assay [21] for quantification of methylated target template. The total quantification assay was composed of two methylation-unspecific oligonucleotides and a scorpion primer unspecific for DNA methylation. The methylation quantification assay (HM assay) uses two methylation-unspecific primers, two methylation-specific blockers (one for each primer) and a scorpion primer specific for methylated DNA. The PCR were done in 20 μl volumes (1 × QuantiTect Multiplex PCR NoROX Kit [Qiagen], DNA [0.25 μl DNA from fresh-frozen and 1 μl from Saccomanno-fixed specimens] and oligonucleotides [Table 2]).

**Table 2:** Oligonucleotide specifications.

Concentration [μM]		
	
Total Quantification Assay	Methylation Quantification Assay	**Sequence (5' **→ **3')**
0.3	0.5	GTTTTTTGGATAGTTAGGTAAT
0.15	0.15	CCTCCTACCTTCTAACCC
0.15	NA	6-FAM-CCGGGGTTGTATGAGTATAGGCCCCGG-BHQ1-C18-CCTCCTACCTTCTAACCC
NA	0.15	6-FAM-CCGGGGTTGTATGAGTATAGGCCCCGG-BHQ1-C18-CCTCCTACCTTCTAACCC
NA	1	TAATTTTTGTTTTGTTTGTTTGATTGGGGTTGTATGA-SpacerC3
NA	1	ACCCAACTTAAACAACAAACCCTTTA-SpacerC3

All oligonucleotides were supplied from biomers.net GmbH, Ulm, Germany.

PCR were performed using a 7900HT Fast Real-Time PCR (Applied Biosystems, CA, USA) using the following temperature profile: 15 min/95°C and 45 cycles with 15s/95°C and 30s/58°C.

### Data and Statistical Analysis

For each sample a relative methylation value was determined using the ΔΔCT method [22,23] as follows: ΔΔCT_Sample _= ΔCT_Sample _- CT_Calibrator_, where ΔCT_Sample _= CT_Sample/Total Quantification Assay _- CT_Sample/Methylation Quantification Assay _and ΔCT_Calibrator _= CT_Calibrator/Total Quantification Assay _- CT_Sample/Methylation Quantification Assay_. ΔΔCTs were measured in triplicates. Sample quality acceptance criterion: Samples were excluded from the study when CT_Sample/Total Quantification Assay >_ (CT_Calibrator/Total Quantification Assay _+ 4). Since the sample contained 50 ng total DNA, this excludes samples with less than approximately 3 ng input DNA into the PCR when assuming a PCR efficiency of 100%.

A methylation cut-off was assigned for dichotomization of the methylation value. Samples having a ΔΔCT value above the cut-off were labeled positive, all others were negative. The cut-off was chosen to reduce the false positive rate to less than 5% for benign samples.

The performance of the assay was reported by means of sensitivity and specificity. Sensitivity is defined as the ratio of correctly assigned positive lung cancer samples in all lung cancer samples. Specificity is defined as the ratio of correctly assigned negative samples in all normal/benign lung samples. Sensitivity and specificity estimates are reported as frequency estimates with 95% confidence intervals based on binomial distributions.

## Results

**Figure 1:**
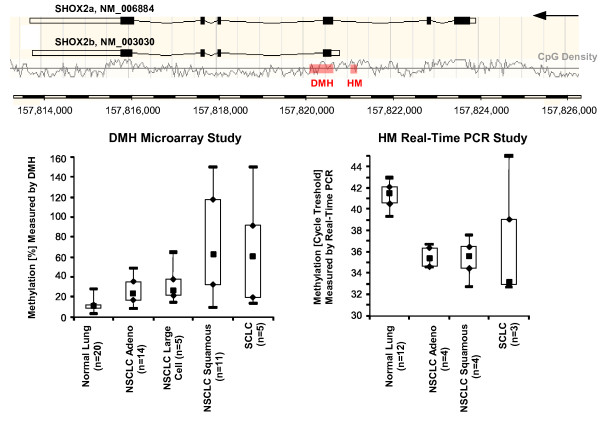
**Location and results of the DMH and real-time PCR assays used for discovery and confirmation of SHOX2 DNA methylation as a lung cancer biomarker**. Tissue samples from 20 normal lungs and 35 lung tumors (14 adenocarcinoma, 11 squamous, 5 large, and 5 small cell lung carcinoma) were analyzed by DMH. Results were confirmed using a SYBR Green HM real-time PCR assay.

Lung tumor specimens from 35 patients (14 adenocarcinoma, 11 squamous, 5 large, and 5 small cell lung carcinoma) and 20 normal lung tissue samples were analyzed using DMH technology, a method for genome-wide methylation profiling. DNA methylation of SHOX2 was identified as a biomarker capable of differentiating between lung cancer tissues and normal tissues (t-test p-value = 0.0003, Wilcoxon Rank-Sum test p-value = 0.0006). A SYBR Green real-time PCR assay, located in close proximity to the SHOX2 DMH amplicon, was designed to confirm the findings from the DMH analysis. Left-over DNA for SYBR Green real-time PCR analysis was available from 12 normal lung tissues and 11 lung cancer tissues (4 adenocarcinoma, 4 squamous, and 3 small cell lung carcinoma). Figure 1 shows the results of the analyses and location of the DMH and the real-time PCR amplicon. Both assays are located in a CpG-rich region around the transcription start site of the b variant of SHOX2 (SHOX2b, NM_003030). Ten out of 11 tumor tissues showed higher methylation of the SHOX2 gene as compared to the normal lung tissues indicated by lower CT values (t-test p-value < 0.0001, Wilcoxon Rank-Sum test p-value = 0.0044). One tumor showed no SHOX2 DNA methylation. The highest methylation levels were found in small cell and squamous cell carcinomas.

**Figure 2:**
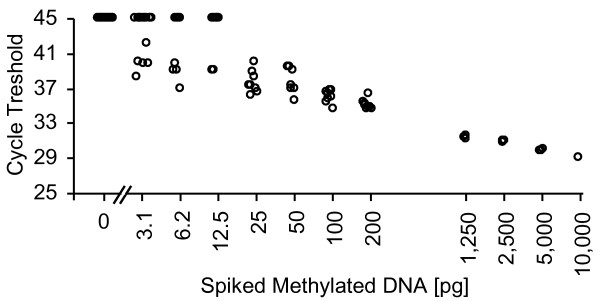
**Analytical assay performance. **Analytical performance of the quantitative real time PCR assay for quantifying SHOX2 DNA methylation. Different amounts of methylated DNA (3.1 - 10,000 pg) were spiked into a background of 50,000 pg unmethylated DNA. Number of replicates: Sixteen (0 and 3.1 pg), eight (6.2-200 pg) and three (1,250-10,000), respectively.

A real time PCR assay for the relative and sensitive detection of methylated SHOX2 DNA in a background of high amounts of unmethylated DNA was developed. The technical performance of the assay is shown in Figure 2. Different amounts (3.1 - 10,000 pg) of bisulfite converted artificially methylated DNA were spiked into a background of 50,000 pg unmethylated DNA from sperm in order to characterize the performance of the assays. The amount of 3.1 - 10,000 pg methylated DNA correspond to the DNA content from approximately 0.5 - 1,600 diploid cells. The assay allowed for the reliable detection of 25 pg (≈ four diploid cells) of methylated DNA in a background of 50,000 pg (≈ 8,000 diploid cells) unmethylated DNA, respectively. Lower amounts of methylated DNA are sporadically detected as expected due to statistical reasons when analyzing single copies of DNA.

**Figure 3:**
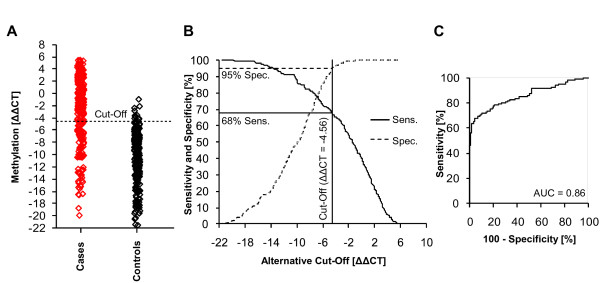
**Clinical performance of the SHOX2 DNA methylation biomarker**. Valid measurements were obtained from 523 patients (281 cases, 242 controls). A: SHOX2 DNA methylation values measured in cases (red) and controls (black). Low ΔΔCT indicate a low SHOX2 DNA methylation. A ΔΔCT = 0 refers to a sample showing the same methylation as the calibrator DNA (1%). B: Resulting sensitivity and specificity when using alternative cut-offs for patient stratification. C: Receiver Operating Characteristic (ROC) and the resulting Area Under the Curve (AUC).

The assay was used to quantify the DNA methylation in 634 fresh-frozen and Saccomanno-fixed bronchial aspirate samples. 111 samples (68 Saccomanno-fixed cases, 4 fresh-frozen cases, 34 Saccomanno-fixed controls, 5 fresh-frozen controls) failed the sample quality control because their DNA yield was too low. These were excluded from analysis. The results of the remaining 523 patient samples are shown in Table 3 and Figure 3. Background DNA methylation of the SHOX2 gene was found in most of the samples necessitating the implementation of the clinical cut-off to dichotomize the quantitative methylation value into a qualitative result (test negative or test positive, Figure 3A). Using a cut-off of ΔΔCT = -4.56, which corresponds to approximately 0.04% methylation, allowed for detection in 68% of cancer patients with high specificity (95%). Applying lower cut-offs for patients stratification led to an increasing sensitivity at decreasing specificity (Figure 3B). The resulting AUC of the ROC was 0.86 (Figure 3C).

**Table 3:** Subtype analysis.

	Histology					
	
Tumor Stage	Adeno-carcinoma	Squamous Cell Carcinoma	NSCLC NOS	SCLC	Other/Unknown	All
**Stage I**	10/32 (31%)	19/24 (79%)	3/3 (100%)	-/-	-/-	32/59 (54%)
**Stage II**	7/17 (41%)	20/21 (95%)	2/3 (67%)	2/2 (100%)	-/-	31/43 (72%)
**Stage III**	20/34 (59%)	36/45 (80%)	16/22 (73%)	6/6 (100%)	1/1 (100%)	79/108 (73%)
**Stage IV**	12/23 (52%)	6/9 (67%)	4/8 (50%)	20/21 (95%)	0/1 (0%)	42/62 (68%)
**Stage unknown**	2/3 (67%)	3/4 (75%)	1/1 (100%)	-/-	0/1 (0%)	6/9 (67%)
**All**	51/109 (47%)	84/103 (82%)	26/37 (70%)	28/29 (97%)	1/3 (33%)	190/281 (68%)

Stage and histology specific performance of the SHOX2 DNA methylation biomarker in 523 bronchial aspirates from patients with suspected lung cancer.

The performance of the SHOX2 biomarker was further investigated with respect to the histological subtype (adenocarcinoma, squamous cell carcinoma, other) and the stage (I-IV, UICC, Table 3). The sensitivity slightly increased at a higher stage. Overall lower sensitivity was found for adenocarcinoma as compared to the other subtypes. The tumor marker performance was notably good in the subgroups of squamous cell carcinoma and SCLC, with sensitivities of 82% and 97%, respectively. This is in concordance with the results from the DMH study where overall higher methylation was found in squamous cell carcinoma and SCLC (Figure 1).

Cytological results were available from 162 patient samples of which 156 samples passed the quality criterion. An analysis with regard to cytology (Table 4) revealed that SHOX2 DNA methylation made it possible to identify 62% of cases which were classified as cytologically negative.

**Table 4:** Clinical performance of the SHOX2 DNA methylation biomarker with regards to cytology (only from 523 valid samples passing the quality criterion).

Cytology Result	Cases Total	Controls Total	Test Positive Cases (Sensitivity [%])	Test Negative Controls (Specificity [%])
All	281	242	190 (68%)	230 (95%)
Unknown	187	180	128 (68%)	171 (95%)
Negative	73	62	45 (62%)	59 (95%)
Suspicious	15	0	12 (80%)	-
Positive	6	0	5 (83%)	-

Among these 523 patients, cytological results were available from 156 patient samples (62 negative controls, 73 negative, 15 suspicious, and 6 positive cases).

Methylation values and detailed clinical data for all patients can be found in Additional file 1: Patient data overview.

## Discussion

DNA methylation has been shown to play an important role in carcinogenesis [15] and DNA methylation alterations are therefore among the most promising candidates in biomarker research. Several previous studies specifically targeted DNA methylation biomarkers for their potential to improve clinical lung cancer management [9-11,24,25].

The objective of this study was to show that SHOX2 DNA methylation is a useful tumor marker to aid the diagnostic workup for suspected lung cancer. The objective of this workup is to diagnose and stage patients using the safest, least invasive and affordable method [26]. In today's clinical practice, a variety of procedures are combined for this purpose. However, due to their shortcomings, e.g. the invasiveness or the limitations in diagnostic performance [27], there is a need for improvement by additional diagnostic tools. One way of achieving this goal is to use tumor markers providing additional information based on existent material yielded from diagnostic procedures.

The most appropriate sample for biomarker studies in lung cancer is bronchial aspirate because of its general availability in routine clinical practice. Bronchial fluid or alternatively bronchial brushings are collected with little risk and extra effort during the first bronchoscopy, which is performed as an integral part in the diagnostic workup.

The material is derived predominantly from the circumscribed clinical region of interest with little contamination from other parts of the body. After cytological analysis by a pathologist, there is usually sufficient leftover material to extract DNA for methylation analysis.

Despite many studies showing a clinical value of sputum samples [28,29], its use has not been widely adopted in clinical routine, which thus limits their utility. A similar situation is also seen in blood, where the total amount of lung derived DNA and the fraction of tumor DNA contained in a sample are expected to be lower than in aspirates. In addition, blood plasma contains a complex mixture of DNA originating potentially from any part of the body. Other tumors, e.g. colon tumors, will probably release tumor cells and tumor DNA into the blood stream as well. In contrast, tumor cells and tumor DNA found in the lungs are most likely from lung tumors or lung metastases and therefore lead to an increased specificity for lung cancer due to the choice of a lung specific analyte. Epigenetic inactivation of tumor supressor genes is critical to the pathogenesis of cancers and some DNA methylation biomarkers, e.g. RASSF1A, are known to be methylated in several different tumor types, i.e. lung, breast, prostate, glioma, neuroblastoma and kidney cancer (for review: [30,31]). Thus, the biological marker requirements for analysing blood are higher and the markers need to be truly specific for lung tumor DNA to ensure a highly specific lung cancer test.

One of the most common fixatives used for aspirates in clinical practice is Saccomanno's reagent. It allows for the storage of samples at ambient temperature for several years, thereby maintaining morphological characteristics and is known to preserve DNA and has been previously shown to be suitable for molecular biological analysis [32,33]. The combination of the characteristics described above makes bronchial aspirates a preferred choice of material for developing a diagnostic test for lung cancer based on DNA methylation.

In this study, DNA methylation of SHOX2 was found as a highly accurate tumor marker for identifying patients with lung cancer based on the analysis of bronchial aspirates. The hypermethylation of SHOX2 in lung cancer tissue has otherwise not been described in the literature so far. The human homeobox gene SHOX2 (short stature homeobox 2, formerly SHOT) is located on the long arm of chromosome 3 (3q25-q26.1). The gene - approx. 10 kbp in size - is known to be transcribed in two different isoforms, SHOX2a (993 bp) and SHOX2b (570 bp) [34], but additional protein-encoding splice variants may exist (Genbank information). Within the SHOX2 gene, two large CpG islands could be identified, with one island covering 1 kbp in the 5'-region and one 0.5 kbp island in the 3'-region of the gene. So far, methylation status and impact of these CpG islands on SHOX2 transcription is not known. Homeobox genes code for proteins harbouring specific DNA-binding homeodomains (homeoproteins). They play fundamental roles in vertebrate development and differentiation by acting as transcriptional regulators. Expression of homeobox proteins themselves is controlled both on the transcriptional and translational level. SHOX2 is a known regulator of chondrocyte hypertrophy and has important functions in skeleton development and embryogenic pattern formation [35]. Other regulatory functions affect embryonic morphogenesis, heart and nervous system development [34]. Although most of its known functions are linked to early events in human development, SHOX2 seems to be widely expressed in different organs and tissues. Interestingly, SHOX2 expression is frequent in various different types of tumors, among them neuroblastomas [36], breast cancer [37] and squamous cell carcinomas of the lung (Genbank information). Homeoproteins are often found to be deregulated in cancer and both down- and up-regulation can be linked with tumor development and progression by activating or repressing multiple downstream genes, thereby acting as proto-oncogenes or tumor suppressor genes [38-40]. However, a direct or indirect implication of SHOX2 as transcriptional regulator during cancerogenesis can be hypothesized.

A significant number of samples analysed in this study were selected according to an inconclusive (negative) cytology result. SHOX2 DNA methylation allowed for an accurate detection of lung cancer patients even in this group of cytologically negative patient samples. In clinical practice, the first bronchoscopy has been found to identify less than half of the lung cancer patients [2]. Based on these results, the use of the SHOX2 tumor marker in a confirmatory test for the diagnosis of lung cancer can be expected to identify more than half of the remaining lung cancer patients in this population. This methylation assay may potentially speed up and simplify the workup for test positive patients by reducing the need for additional diagnostic procedures.

The SHOX2 methylation level in bronchial aspirates from patients with stage I disease was found to be lower than from patients with more advanced malignant disease leading to a lower sensitivity for stage I patients. The most likely explanation is the smaller size of the tumor might result in less malignant cells in the corresponding bronchial aspirate; other confounding factors like tumor aggressiveness and the relation of the tumor to the bronchial system need to be considered as well. Possible explanations are that slower growing tumors are over-represented in this population, because they are more likely to be clinically detected at an early stage and they shed less DNA into the bronchial system due to their lower aggressiveness.

The investigation of the impact of histological lung cancer subtypes on the methylation levels of aspirates showed that patients with SCLC and NSCLC squamous cell carcinoma have higher levels of methylation than patients with adenocarcinoma. There is no obvious explanation for this phenomenon, but the observation is in line with other studies [11], which showed a lower sensitivity of marker panels in adenocarcinoma compared to squamous cell NSCLC. A confounding effect of the tumor location needs to be considered as centrally located squamous cell carcinomas are usually easier to assess via bronchoscope, which makes them likely to yield more target DNA compared to a peripheral location. The rationale and the impact of performance differences of histological subtypes require further investigation.

Ideally, a tumor marker would detect all stages and histological subtypes equally well. Nevertheless, in today's clinical practice the vast majority of patients are diagnosed with advanced stage disease and a test to diagnose these patients accurately represents a medical need, making SHOX2 a clinically useful marker. Therefore, such assay is proposed as a useful tool for confirming the presence of malignant lung disease in patients with suspected lung cancer, especially when the histology and cytology results from specimen obtained by bronchoscopy do not confirm the presence of malignant lung disease. The assay is currently translated into an CE marked IVD test for patients undergoing first-time bronchoscopy for suspected lung cancer. The test result will be suited for use by physicians as an aid in diagnosis of lung cancer adjunct to existing clinical and pathological information. A validation study with an independent patient population is ongoing.

## Conclusions

Hypermethylation of SHOX2 in bronchial aspirates is a sensitive and specific biomarker for identifying subjects with lung carcinoma, especially if histological and cytological findings after bronchoscopy are ambiguous.

## Abbreviations

AUC: Area Under the Curve; CE: Conformité Européenne; CI: Confidence Interval; CT: Cycle Treshold; DMH: Differential Methylation Hybridization; HM: Heavy Methyl; IVD: In Vitro Diagnostics; NOS: Not Otherwise Specified; NSCLC: Non Small Cell Lung Cancer; PET: Positron Emission Tomography; ROC: Receiver Operating Characteristic; SCLC: Small Cell Lung Cancer; SHOX2: Short Stature Homeobox 2; UICC: Union Internationale Contre le Cancer;

## Competing interests

Volker Liebenberg, Dimo Dietrich, Thomas Schlegel, Christoph Kneip, Anke Seegebarth, Nadja Flemming, Stefanie Seemann, Jörn Lewin, Juergen Distler, Ulrike Wille, and Reimo Tetzner are or have been employees and/or stockholders of Epigenomics AG, a company that aims to commercialize DNA methylation markers. John Field is a member of the Epigenomics Advisory Board.

## Authors' contributions

BS, VL, DD, MF, OR, and JKF participated in the design of the study and its supervision and drafted the manuscript. DD and CK designed and developed the assay, coordinated the study and drafted the manuscript. AS carried out the experiments. TS carried out the statistical analysis. SW, TL, MW, CW, OR, and JKF participated in the design of the study and collected and characterized the sample material. NF, UW and SS participated in the development of the assay. RT, JL and JD participated in marker identification. All authors read and approved the final version of the manuscript.

## Pre-publication history

The pre-publication history for this paper can be accessed here:

http://www.biomedcentral.com/1471-2407/10/600/prepub

## Supplementary Material

Additional file 1 **Patient information and SHOX2 DNA methylation data**. This excel spreadsheet (.xls) contains the relevant clinical information (i.e. age, gender, smoking habits, diagnosis, sample type) and the measured SHOX2 methylation values for each patient. Click here for file
